# An artificial neural network approach integrating plasma proteomics and genetic data identifies *PLXNA4* as a new susceptibility locus for pulmonary embolism

**DOI:** 10.1038/s41598-021-93390-7

**Published:** 2021-07-07

**Authors:** Misbah Razzaq, Maria Jesus Iglesias, Manal Ibrahim-Kosta, Louisa Goumidi, Omar Soukarieh, Carole Proust, Maguelonne Roux, Pierre Suchon, Anne Boland, Delphine Daiain, Robert Olaso, Sebastian Havervall, Charlotte Thalin, Lynn Butler, Jean-François Deleuze, Jacob Odeberg, Pierre-Emmanuel Morange, David-Alexandre Trégouët

**Affiliations:** 1 INSERM, BPH, U1219, Université Bordeaux, 33000 Bordeaux, France; 2 Laboratory of Excellence GENMED (Medical Genomics), Strasbourg, France; 3 Science for Life Laboratory, Department of Protein Science, CBH, KTH Royal Institute of Technology, Stockholm, Sweden; 4 Department of Clinical Medicine, Faculty of Health Science, The Arctic University of Tromsö, Tromsö, Norway; 5 INSERM, INRAE, C2VN, Aix Marseille University, Marseille, France; 6 Hematology Laboratory, La Timone University Hospital of Marseille, Marseille, France; 7 CEA, Centre National de Recherche en Génomique Humaine, Université Paris-Saclay, 91057 Evry, France; 8 Division of Internal Medicine, Department of Clinical Sciences, Karolinska Institutet, Danderyd Hospital, Stockholm, Sweden; 9 Clinical Chemistry and Blood Coagulation Research, Department of Molecular Medicine and Surgery, Karolinska Institute, Karolinska University Hospital, 171 76 Stockholm, Sweden; 10 Centre D’Etude du Polymorphisme Humain, Fondation Jean Dausset, Paris, France

**Keywords:** Data integration, Genetics, Machine learning

## Abstract

Venous thromboembolism is the third common cardiovascular disease and is composed of two entities, deep vein thrombosis (DVT) and its potential fatal form, pulmonary embolism (PE). While PE is observed in ~ 40% of patients with documented DVT, there is limited biomarkers that can help identifying patients at high PE risk. To fill this need, we implemented a two hidden-layers artificial neural networks (ANN) on 376 antibodies and 19 biological traits measured in the plasma of 1388 DVT patients, with or without PE, of the MARTHA study. We used the LIME algorithm to obtain a linear approximate of the resulting ANN prediction model. As MARTHA patients were typed for genotyping DNA arrays, a genome wide association study (GWAS) was conducted on the LIME estimate. Detected single nucleotide polymorphisms (SNPs) were tested for association with PE risk in MARTHA. Main findings were replicated in the EOVT study composed of 143 PE patients and 196 DVT only patients. The derived ANN model for PE achieved an accuracy of 0.89 and 0.79 in our training and testing sets, respectively. A GWAS on the LIME approximate identified a strong statistical association peak (rs1424597: p = 5.3 × 10^–7^) at the *PLXNA4* locus. Homozygote carriers for the rs1424597-A allele were then more frequently observed in PE than in DVT patients from the MARTHA (2% vs. 0.4%, p = 0.005) and the EOVT (3% vs. 0%, p = 0.013) studies. In a sample of 112 COVID-19 patients known to have endotheliopathy leading to acute lung injury and an increased risk of PE, decreased PLXNA4 levels were associated (p = 0.025) with worsened respiratory function. Using an original integrated proteomics and genetics strategy, we identified *PLXNA4* as a new susceptibility gene for PE whose exact role now needs to be further elucidated.

## Introduction

Deep vein thrombosis (DVT) and pulmonary embolism (PE) are often considered as two sides of the same coin, venous thromboembolism (VTE), the third most common cardiovascular disease. VTE is a complex disease resulting from the interplay of various factors including (epi-)genetics and environmental sources. VTE incidence is estimated at 1 per 1000 patient-years, and its fatal form, PE, is associated with a mortality rate of 6% in the acute phase and 20% after one year^1^. PE generally results from the migration of a blood clot from a deep vein to the lung and is observed in ~ 40% of patients with documented DVT^2^. However, isolated PE without any trace of DVT can also be observed either when the clot has completely migrated to the lung or when it is a pulmonary clot in situ as recently highlighted in COVID-19 patients^3,4^. Even though some specific risk factors for PE have been identified in DVT patients such as obesity, sickle cell disease^5^ as well as some genetic variations in *F5*^5^ and *GRK5*^6^ genes, the exact, likely multifactorial, biological mechanisms that lead to PE are still not fully characterized. Besides, there are still limited biomarkers that can help discriminating patients that will develop PE from those who will not, the former being then at higher risk of death. Thus, there is clearly a need for novel PE-associated molecular markers to be identified.

Plasma is an ideal potential source for VTE biomarkers; the intravascular compartment itself is the site of disease manifestation and tests are relatively non-invasive, quick and cheap. Several types of molecular determinants can be assessed in plasma samples including microRNAs, metabolites and proteins, and all of them have been investigated in the context of VTE. For example, plasma microRNAs have been assessed in relation to VTE recurrence^7,8^. Plasma proteomics has been employed to discover novel proteins associated with VTE risk^9,10^ and plasma metabolomics used to identify novel mechanisms involved in VTE etiology^11,12^. Only one study has so far adopted an exploratory plasma proteomics strategy to identify novel proteins associated with high-risk versus low-risk of PE in humans. This study^13^ was based on a relatively small sample size and compared 6 patients with high risk of PE to 6 patients at low PE risk, risk being classified based on clinical presentations and symptoms, with plasma samples profiled by matrix-assisted laser desorption/ionization–time-of-flight/time-of-flight mass spectrometry (MALDI-TOF/TOF MS).

**Figure 1:**
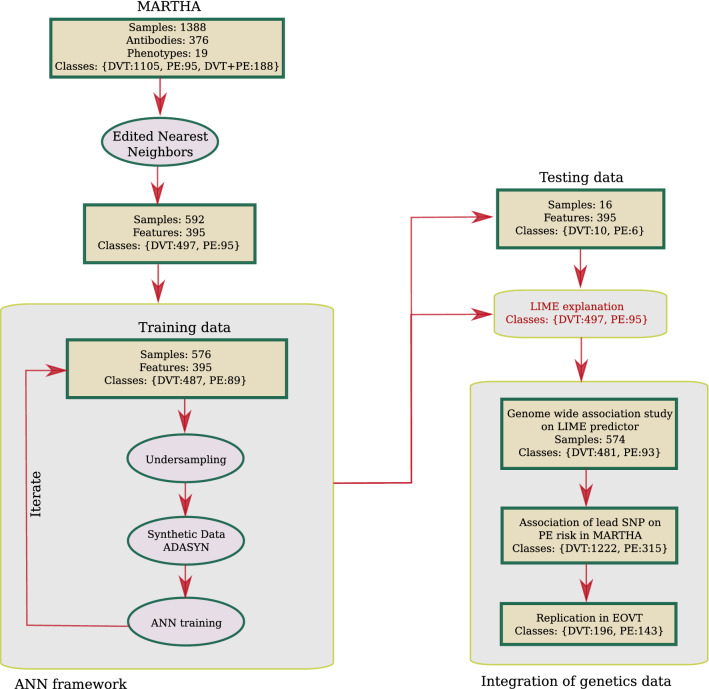
Analysis workflow of the present study.

In this work, we aim at identifying novel molecular phenotypes that could help in better characterizing the biological mechanisms involved in the development of PE in VTE patients. For this, 234 plasma proteins targeted with 376 protein specific antibodies, with the major part derived from the Human Protein Atlas (HPA) repository^14^ were profiled in 1388 VTE patients selected from the MARTHA study^15,16^ and from whom 283 had experienced a symptomatic PE event. To explore far beyond the search for linear associations between protein levels and PE risk and to identify more complex relationships that could serve as integrative markers of upstream/downstream mechanisms involving molecular determinants that have not necessarily been measured, we deployed a sequential procedure implementing several methodologies selected from the machine-learning domain. Briefly, and as summarized in Fig. 1 and more detailed thereafter, the first step consists in applying an under-sampling algorithm (edited nearest neighbors)^17^ to remove individuals with strong data heterogeneity that would hamper the efficiency of the downstream analyses, leaving to subsample of 592 VTE patients (497 DVT and 95 PE). This subsample was then used in an Artificial Neural Network (ANN) learning framework in order to predict PE from proteomics and clinical data. We then used the Local Interpretable Model-agnostic Explanations (LIME) algorithm^18^ to derive a linear approximate of the ANN based predictor for PE risk which would, in addition, have a more meaningful biological interpretation. As MARTHA patients have been previously typed for genome-wide genotype data, we then conducted a genome wide association study of the LIME predictor of PE in order to detect single nucleotide polymorphisms (SNPs) associated with the predictor with the hope that the integration of genetic and proteomic data could provide additional insights into the pathophysiology underlying the identified predictor^19,20^. SNPs with strong statistical association with the LIME predictor were tested for association with PE risk in the whole original MARTHA dataset and significant associations were further tested for replication in an independent study of 339 VTE patients including 143 with PE. Sequencing data were also scrutinized in some patients with observed VTE outcomes poorly predicted by our ANN/LIME prediction models in order to identify rare variants that could be responsible for the observed phenotypes.

## Materials and methods

### Ethical approval

Each individual study on which the work is based was approved by its institutional ethics committee and informed written consent was obtained in accordance with the Declaration of Helsinki. Ethics approval were obtained from the “Departement santé de la direction générale de la recherche et de l’innovation du ministère” (Projects DC: 2008-880 and 09.576) and from the institutional ethics committees of the Kremlin-Bicetre Hospital.

### MARTHA study

The MARTHA population is composed of VTE patients recruited from the Thrombophilia center of La Timone hospital (Marseille, France) and free of any chronic conditions and of any well characterized genetic risk factors including antithrombin, protein C or protein S deficiency, homozygosity for FV Leiden or Factor II 20210A, and lupus anticoagulant. Detailed description of the MARTHA population has been provided elsewhere^15,21^.

#### MARTHA proteomics substudy

A sample of 1388 MARTHA patients with available plasma samples were profiled for targeted plasma proteomic investigations as described below.

*MARTHA genetic substudy*. From the whole MARTHA population, 1592 patients with DNA available were genotyped with high-throughput genotyping arrays (see below).

Patients were also phenotyped for 19 quantitative traits known to be involved in thrombotic biological processes (Supplementary Table 1).

### Plasma proteomic profiling

#### Generation of antibody suspension bead array (SBA)

The multiplex antibody suspension bead array (SBA) was created by covalent coupling of 339 Human Protein Atlas (HPA) antibodies, 13 from commercial providers and 25 monoclonal BSI antibodies (BioSystems International Kft) targeting 234 unique candidate proteins (Supplemental Table 2). These proteins were selected for (1) their known roles in the coagulation/fibrinolysis cascade and/or intermediate traits of relevance to thrombosis, (2) their specific expression in endothelial cells (a key cell type involved in thrombosis physiopathology) or (3) encoded by genes identified in pangenomic studies as associated with several cardiovascular disease-linked biological pathways (e.g. platelet function, renal function, inflammation).

Antibodies were individually coupled to carboxylated magnetic beads (MagPlex-C, Luminex Corp.) generating up to 384 different bead identities (IDs), essentially according to methods previously described^9,22^. The final multiplexed suspension bead array was prepared by combining all 384 antibody coupled beads into a single SBA stock with a concentration of approximately 25–40 beads of each antibody bead ID/ul.

#### Plasma labelling and protein profiling assay

Plasma samples were diluted 1:10 in filtered 1xPBS and labelled with biotin (NHS-PEG4-Biotin, Thermo Scientific) for 2 h at 4 °C. The labelling process was terminated by the addition of 12,5ul of 0.5 M HCl pH:8.0 to each sample for 20 min and consecutively storage at − 20 °C until usage^22^. Labelled plasma samples were diluted 1:50 in PVX casein buffer + 10% (v/v) rabbit IgG (0.1% casein, 0.5% polyvinyl alcohol, 0.8% polyvinylpyrrolidone, prepared in 1xPBS). Diluted samples were heat-induced to achieve epitope retrieval for 30 min at 56 °C. Five microliters of the SBA were mixed with 45ul of heat-treated samples for 16–18 h, at RT and constant shake. Unbound complexes were removed by 2 consecutive washes with PBS-T and antibody-bound complexes were cross-linked by resuspending the beads in 0.4% PFA-PBS for 10 min. R-phycoerythrin-conjugated streptavidin (1:750, PBS-T; Invitrogen) was added to all samples for 30 min followed by 2 times washes. Relative amount of each protein complex was expressed as median of fluorescence intensity (MFI) by read out on a FlexMAP3D.

### The early onset venous thrombosis (EOVT) study

This study is composed of 339 VTE patients with documented idiopathic isolated PE or DVT selected according to the same criteria as the MARTHA participants, with the exception that the age of VTE onset was below 50 yrs. Brief characteristics of the EOVT participants are shown in Supplementary Table 3 while detailed description can be found in^21,23^.

### Machine-learning framework for identifying a molecular predictor of PE risk

#### Step 1: Normalization

First, all HPA variables and biological traits were normalized and scaled to have 0 mean and 1 variance to avoid major artificial influence of variables with large range of variations.

#### Step2: Edited nearest neighbors

As our aim was to identify new molecular markers associated with PE, we hypothesized that conducting our discovery phase on isolated PE, an expected less heterogeneous class of VTE patients than the class of patients with both DVT and PE, will increase our chance to identify novel relevant molecular players. As a consequence, we decided to build our ANN model only on patients with isolated PE (N = 95) or with DVT (N = 1105). However, due to the imbalance nature of this dataset with ~ 10 more samples in the DVT class than in the PE class, we applied the edited nearest neighbors (ENN) algorithm, an under sampling method usually used in the field of pattern recognition or classification in presence of unbalanced samples^17^. This method relies on under sampling unit of analysis, in our case individuals, from the majority class by removing the most heterogeneous units. It consists in computing the Euclidean distance between each pair of individuals from their proteomics and biological data and to remove samples whose clinical phenotype (here DVT) is not consistent with that of his/her k nearest neighbors (k = 3 in this work). This led us to the selection of the so called ANN dataset composed of N = 497 DVT and N = 95 PE patients for building our ANN model.

#### Step3: Derivation of an ANN model for PE prediction

To build our ANN model, the ANN dataset was divided into a training set composed of 576 patients (487 DVT and 89 PE) and a testing set of 16 patients (10 DVT and 6 PE), the latter being used for testing the accuracy of the ANN model derived from the former. This allocation was chosen so that the number of PE cases used for training was sufficiently large.

Because the application of a standard ANN methodology to our training set would lead to unstable network for predicting PE due to the imbalance nature of the input data with ~ 5 times more DVT than PE patients, an interactive ANN framework was adopted:

At each iteration *i*,

- A random sample of 30 PE patients and 100 DVT patients is selected from the training set and a sample of 70 synthetic PE samples are generated using the ADASYN algorithm^24^. ADASYN is an adaptive synthetic data generation method where new samples are generated based on the weighted distribution for minority class samples with two main advantages, resolving data imbalance and forcing classifiers to be more sensitive to the minority class. This strategy led to a balanced dataset D*i* of 100 PE and 100 DVT (synthetic) patients on which a ANN is built.
- Using the Di dataset further splitted randomly into 90%/10% training/testing subsamples, a two hidden-layers feed forward neural network was implemented (see Supplementary data for an illustration of the neural network’s structure). In addition to the input layer corresponding to the number of proteomic and biological variables (n = 395), the proposed neural network included a first hidden layer with 395 neurons, a second layer with 128 neurons and an output layer with 2 neurons, representing the DVT and PE classes respectively. The number of neurons were selected by trial and error approach under the constraint that the number of neurons shall be smaller or equal to the number of input variables and higher than the number of output classes.

The Rectified Linear Unit (ReLU) function^25^ was used to activate hidden layers while the softmax activation function^26^was used to generate class probabilities in the output layer.

After fixing the number of nodes, layer and activation function, the process of training the neural network can start. Starting from random weights, forward propagation is used to generate the output of all nodes at all layers while moving from the input to the output layers. The generated final output is compared to the observed class phenotype and an error is calculated using the cross-entropy function^27^. Iteratively, this error was then back-propagated using a gradient descent algorithm^28^ (with learning of 0.01 and batch size of 32) to update weights according to their contribution to the error. In order to reduce over-fitting and obtain the best performing model, the callback feature proposed by the Keras open-source library (https://keras.io/) was employed.

#### Step4: Local interpretable model-agnostic explanations (LIME)

As a neural network is often considered a black box without telling much about which, and how, input variables contribute to the prediction, the LIME methodology^18^ was applied to the final ANN model obtained at Step 3 in order to inform about which input variables (i.e. plasma protein levels) contribute to PE risk prediction and what are the relative weights using a linear approximation of the ANN model.

### Genome wide genotyping

As previously described^16,21^, both MARTHA and EOVT participants have been genotyped with high-density genotyping Illumina arrays and imputed for single nucleotide polymorphisms (SNPs) from the 1000G Phase I Integrated Release Version 2 Haplotypes using MACH (v1.0.18.c) and Minimac (release 2011-10-27) imputation software.

### Genome-Wide Association analysis (GWAS)

Imputed SNPs with imputation quality *r*^2^ greater than 0.5 and with minor allele frequency (MAF) greater than 0.01 were tested for association with the LIME predictor derived in 574 MARTHA participants. Associations with statistical p-value < 5 × 10^–8^ were considered as genome-wide significant.

### Genetic Association Analysis with PE risk

The candidate SNP identified from the GWAS on the LIME predictor was tested for its association with PE risk, both in MARTHA and EOVT participants. For this, we employed the Cochran-Armitage trend for association applied to the best guessed genotypes inferred from the imputed allele dosage at the SNP of interest. Logistic regression was also employed to estimate genetic effects adjusted for age and sex.

### Annotation of the candidate SNP

Identified SNP was examined for association with the expression of its structural gene via publicly available genome-wide gene expression data from multiple cell lines and tissues incl.^29–32^. The GTEx portal (https://gtexportal.org/) was used to investigate the SNPs effect on gene expression. Association of the SNPs with DNA methylation levels from peripheral blood DNA was also investigated using latest results from the GoDMC consortium (http://mqtldb.godmc.org.uk/index). Additional online tools were also used to determine if the candidate SNP could be associated with any biological traits (e.g. HaploReg:https://pubs.broadinstitute.org/mammals/haploreg/haploreg.php; GWAS Catalog: https://www.ebi.ac.uk/gwas/; BIG server: http://big.stats.ox.ac.uk/; GRASP server: https://grasp.nhlbi.nih.gov/Overview.aspx; FinnGen repository: https://www.finngen.fi/en/access_results) or with specific regulatory mechanisms (e.g. RegulomeDB: https://www.regulomedb.org/regulome-search/; Trap: http://trap-score.org/).

### Plasma levels of the identified candidate protein in COVID-19 patients

Given that PE is a frequently observed thrombotic complication in COVID-19 patients^33^, we measured plasma levels of the protein encoded by the identified candidate gene in COVID-19 participants of the COMMUNITY study and assessed their associations with pulmonary complications. The *COMMUNITY study—“COVID-19 biomarker and Immunity study*” is a single center study of 112 patients with COVID-19 disease admitted to general wards, intermediate units, or intensive care units at Danderyd Hospital, Stockholm Sweden between April 15th and May 27th 2020. Inclusion was based on a confirmed diagnosis of SARS-CoV2 infection based on reverse‐transcriptase polymerase chain reaction (RT‐PCR) viral RNA detection of nasopharyngeal or oropharyngeal swabs, or clinical presentation with COVID-19 disease. Exclusion criteria were age < 18 years. Patients were followed longitudinally from inclusion, and blood samples for biobanking of plasma samples were collected shortly after hospital admission and every 2–3 days during the hospital stay. Procedures for blood sampling and plasma preparation have been previously described^34^. Demographic data, routine lab results, comorbidity and information and variables reflecting clinical deterioration, including respiratory support were obtained from medical records. Patients were divided into groups based on respiratory support classified at the time of a sample was drawn, classified into a categorical variable ‘Respiratory Index’ or RI, defined as RI = 0 for no respiratory support, RI = 1 for ≤ 5 L of oxygen on nasal cannula or mask, RI = 2 for > 5 L of oxygen on nasal cannula or mask, RI = 3 for noninvasive respiratory support and RI = 4 for intubation. Level of respiratory support and oxygen supplementation were set at the discretion of the treating physician. For the current study, 339 samples collected from 112 patients were available, with at least 2 samples for 71 of the patients (63.4%). Baseline characteristics are shown in Supplementary Table 4.

The HPA antibody HPA052141 targeting the PLXNA4 protein was used to measure PLXNA4 levels in the 339 available samples from the COMMUNITY study, following a similar protein profiling protocol as that described above.

Association of PLXNA4 plasma levels with respiratory index at baseline was tested using linear regression models. To handle multiple time point measurements, association of PLXNA4 plasma levels with RI was further investigated using all available longitudinal measurements using a linear mixed effect model as implemented in the *nlme* R package. Analyses were adjusted for age, sex and body mass index.

### Whole genome sequencing

From the whole MARTHA study, 200 patients had been selected for whole genome sequencing. These patients were selected to have experienced VTE in absence of strong environmental and genetic risk factors. Besides, these patients should have family history of VTE or multiple unprovoked VTE events, such clinical patterns being compatible with the existence of an underlying VTE causing genetic defect. Genomic DNA was extracted from peripheral blood, using the BioRobot EZ1 workstation. The DNA concentration was determined using the Qubit assay kit (Thermofisher). Whole genome sequencing was performed at the Centre National de Recherche en Génomique Humaine (CNRGH, Institut de Biologie François Jacob, Evry, France). After a complete quality control, 1 µg of genomic DNA was used for each sample to prepare a library for whole genome sequencing, using the Illumina TruSeq DNA PCR-Free Library Preparation Kit, according to the manufacturer's instructions. After normalization and quality control, qualified libraries were sequenced on a HiSeqX5 instrument from Illumina (Illumina Inc., CA, USA) using a paired-end 150 bp reads strategy. One lane of HiSeqX5 flow cell was used per sample specific library in order to reach an average sequencing depth of 30× for each sequenced individual. Sequence quality parameters have been assessed throughout the sequencing run and standard bioinformatics analysis of sequencing data was based on the Illumina pipeline to generate FASTQ file for each sample. FastQ sequences were aligned on human genome hg37 using the BWA-mem program^35^. Variant calling was performed using the GATK HaplotypeCaller (GenomeAnalysisTK-v3.3-0, https://software.broadinstitute.org/gatk/documentation/article.php?id=4148). Single-sample gVCFs files were then aggregated using GATK CombineGVCFs and joint genotyping calling performed by GATK GenotypeGVCFs. Recalibration was then conducted on the whole gVCF following GATK guidelines. Following GATK VQSR, we retained single nucleotide variants in the 99.5% tranche sensitivity threshold and indels in the 99% tranche sensitivity threshold for further analysis and annotated them using Annovar^36^.

As a strategy to identify candidate variants that could explain the VTE phenotype in individuals with discordant class prediction, we first prioritized variants that were likely functional (stop loss/stop gain, frameshift, non-synonymous and splicing variants), located in known VTE associated genes (*ABO, ARID4A, C4BPB, EIF5A, F2, F3, F5, F8, F9, F13A1, FGG, GRK5, MPHOSPH9, MAST2, NUGCC, OSMR, PLAT, PLCG2, PLEK1, PROC, PROS1, SCARA5, SERPINC1, SLC44A2, STAB2, STX10, STXBP5, THBD, TSPAN15, VWF*)^37–39^, that have not been reported or at a low frequency (< 1‰) in public genomic data repositories (dbSNP, GnomAD) and that was present in only one of the 200 sequenced patients. If no candidate variants was identified in known VTE genes, we extended our search to whole coding genes and also took into account the predicted deleteriousness of selected candidates using in silico tools such as SIFT, PolyPhen and CADD-v1.2^40^ to further reduce the number of candidates.

## Results

### Data description

The MARTHA proteomics substudy was composed of 1,388 VTE patients among which 1105 were diagnosed for DVT, 95 with isolated PE and 188 with both DVT and PE (Table 1).

**Table 1:** Characteristics of the MARTHA proteomics study.

	DVT	PE	DVT + PE
N	1105	95	188
Age at sampling	46.67 (14.90)	48.63 (15.26)	51.57 (16.99)
Age at first VTE	40.89 (15.28)	41.64 (15.02)	44.22 (17.56)
Female sex	716 (65%)	78 (82%)	112 (60%)
Women under oral contraceptives at VTE event	286 (26%)	35 (37%)	45 (24%)
FV Leiden (rs6025) heterozygotes	255 (23%)	17 (18%)	39 (21%)
Anticoagulant therapy at plasma sampling	303 (27%)	29 (31%)	76 (40%)
Smokers	209 (19%)	18 (19%)	24 (13%)
BMI	25.14 (4.57)	25.20(4.39)	26.43(4.62)

Data shown correspond to mean (standard deviation) and count (percentage) for continuous and categorical variables, respectively.

*DVT* Deep Vein Thrombosis, *PE* Pulmonary Embolism, *BMI* Body Mass Index.

**Figure 2:**
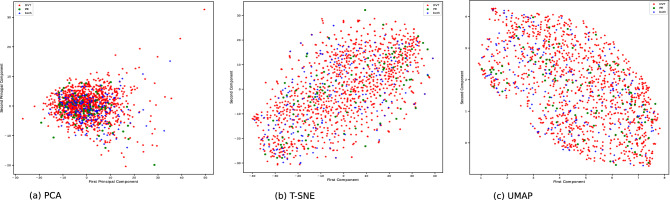
Graphical representation of the HPAs and biological MARTHA data projected on the first two principal components derived from standard principal components analysis (**a**), t-SNE (**b**) and UMAP (**c**) techniques.

Exploration of this dataset using high-dimensional visualization techniques including principal component analysis, t-distributed stochastic neighbor embedding (t-SNE)^41^ and Uniform Manifold Approximation and Projection (UMAP)^42^ did not reveal any specific stratification in the data nor outliers (Fig. 2) but rather illustrates that the three class of patients (DVT, PE, DVT + PE) could not be easily separated.

*Artificial Neural Network for PE*—As the accuracy/efficiency of any ANN strongly depend on the quality/homogeneity of the input data, we first applied the edited nearest neighbors algorithm^17^ to perform under sampling of the majority class (DVT) and obtain a more homogeneous set of DVT patients, and further discarded the DVT + PE class to avoid adding noise in discriminating between PE and non PE patients. This strategy led to the selection of a subsample (referred thereafter to as the ANN dataset) of 592 patients (497 DVT and 95 PE) whose proteomics/biological entered the ANN analysis. Visual inspection of the proteomics and biological data in the selected ANN sample is shown in Supplementary Fig. 2.

A two hidden-layers ANN was then built from the ANN dataset with a training set of 576 patients (487 DVT and 89 PE) and a testing set of 16 patients (10 DVT and 6 PE). This allocation was chosen so that the number of PE cases used for training was sufficiently large. Because the training set presented with a strong imbalance with respect to the DVT/PE classes with ~ 5 times more DVT than PE patients, the ANN was trained iteratively as described in the Materials and Methods section. By completion of the iterative algorithm, the final ANN obtained an area under the operative curve (AUC) of 0.89. Of more interest are the performances of the ANN in the testing set. Indeed, our ANN got F1-scores of 0.82 and 0.60 for the DVT and PE classes, respectively, and a global AUC of 0.79 in the testing set.

We then used the LIME algorithm to obtain a local linear approximate of the ANN predictions. In the testing set, the LIME prediction achieved an overall AUC of 0.77 instead of 0.79 for ANN. For each of the 16 patients in the testing set, we compared the individual predictions of their observed VTE event provided by the ANN and LIME methods (Table 2). In general, ANN and LIME predictions were rather consistent even if the ANN predictions seem to be more accurate in predicting DVT while LIME appears slightly more accurate in predicting PE. The average prediction in correctly classifying DVT patients was 0.872 by ANN compared to 0.748 by LIME. Note that one DVT patient (individual 10) was wrongly predicted to be PE by the ANN predictor, but not by the LIME predictor. Conversely, the average prediction in correctly classifying PE patients was 0.498 by ANN compared to 0.578 by LIME. Two PE patients (individuals 11 and 12) presented low predictions of being PE, using both ANN and LIME predictors.

**Table 2:** Individual predictions of VT event provided by ANN and LIME in the 16 patients of the testing set.

Individual	Observed clinical class	ANN prediction for class PE	Local prediction for class PE
1	DVT	0.04	0.31
2	DVT	0.00	0.18
3	DVT	0.03	0.24
4	DVT	0.02	0.17
5	DVT	0.00	0.23
6	DVT	0.02	0.32
7	DVT	0.00	0.25
8	DVT	0.04	0.22
9	DVT	0.25	0.34
10	DVT	0.88	0.26
11	PE	0.00	0.30
12	PE	0.20	0.31
13	PE	0.98	0.94
14	PE	1.0	1.0
15	PE	0.01	0.15
16	PE	0.80	0.77

**Figure 3:**
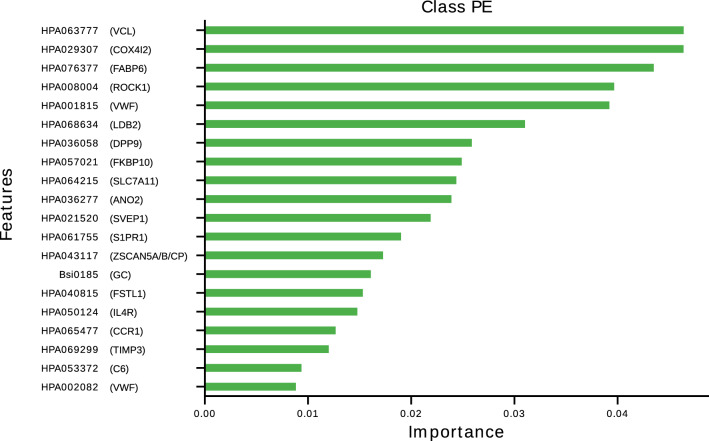
List of the top 20 antibodies contributing the most to the prediction model for PE.

We then assessed the correlation of the LIME predictor with the available biological phenotypes. No strong correlation was observed (Supplementary Table 5). However, the LIME predictor showed marginal positive correlation with fibrinogen (ρ = 0.12, p = 5.7 × 10^–3^) and factor VIII (ρ = 0.16, p = 0.013) plasma levels, and marginal negative correlation with prothrombin time (ρ = −0.10, p = 0.029) and protein S (ρ = −0.10, p = 0.021) plasma levels. To go further into the biological interpretation of the LIME predictor, we sought to identify which proteins contribute the most to the definition of the LIME predictor. Figure 3 display the top 20 most contributing antibodies/proteins. Of note, 5 proteins tended to have substantial more importance than the remaining ones, among which three include proteins that had been selected because their gene expression (COX4I2, VCL, VWF) was found to be specifically enriched in endothelial cells^43^.

### Genetics of the LIME predictor

To get additional information about the biological mechanisms that could underlie the linear LIME predictor, we conducted a GWAS on this predictor considered as a quantitative linear trait in a sample of 574 individuals of the ANN subsample with GWAS data. While no SNP reached genome-wide significance, we observed a peak of strong suggestive statistical association on chromosome 7 at the *PLXNA4* locus (Supplementary Fig. 1–Supplementary Table 6). The sentinel SNP (p = 5.33 × 10^–7^) was rs1424597 whose minor A allele with frequency of 0.09 was associated with an increase of + 0.169 ± 0.034 in LIME predictor values. We then tested the association of the rs1424597 polymorphisms with PE in the whole MARTHA samples. As shown in Table 3, the rs1424597-A allele tended to be more frequent in patients with PE than in patients with DVT only (0.11 vs. 0.08). However, looking deeply to the genotypic distribution revealed a pattern of association more compatible with a recessive effect for the rs1424597-A allele. Carriers of the AA genotype were more frequently observed in the PE than in the DVT groups (2% vs. 0.4%), carrying the AA genotype being associated with a significantly higher risk of PE (OR 5.3 [1.7–17.0], p = 0.005). This pattern of recessive association was also observed in the EOVT study composed of 143 PE patients and 196 DVT patient. In EOVT, 3% of PE patients were carriers of the AA genotype and none of the DVT patients, the Fisher exact test for the recessive A allele effect being significant (p = 0.013). Looking back to the original GWAS results for the LIME predictor revealed that the association of rs1424597 with the predictor was also compatible with a recessive effect. Mean values for the LIME predictor were 0.33 ± 0.31, 0.48 ± 0.36 and 0.79 ± 0.45, in GG, GA and AA genotypes, respectively.

**Table 3:** Association of rs1424597 with PE risk in the MARTHA and EOVT studies.

	MARTHA		EOVT	
	DVT	PE	DVT	PE
GG	1028 (84%)	258 (80%)	149 (76%)	110 (77%)
GA	185 (15%)	59 (18%)	47 (24%)	28 (20%)
AA	5 (< 1%)	7 (2%)	0 (−)	5 (3%)
MAF^1^	0.080	0.113	0.120	0.133
OR^2^	5.338 [1.676–17.00] p = 0.005		Undefined	

*MAF* Minor Allele Frequency.

OR: Allelic Odds Ratio [95% CI] adjusted for sex and age at DVT/PE event, under the assumption of recessive effect.

We then interrogated various public resources (See Methods) to investigate if the *PLXNA4* rs1424597 could be associated with additional clinical or biological traits as well as regulatory mechanisms. The only robust and strong identified association relates to a meta-analysis of whole blood DNA methylation data performed in more than 27,000 individuals part of the GoDMC consortium (https://doi.org/10.1101/2020.09.01.20180406) and where rs1424597 was statistically associated (p = 8.7 × 10^–91^) with methylation levels at the CpG cg06087029 site. The rs1424597-A allele was associated with decreased levels of the cg06087029 site that maps to the *ATRIP* locus on chromosome 3. Noteworthy, the *PLXNA4* polymorphism that associated the most with cg06087029 in GoDMC, rs17219279 (p = 2.7 × 10^–119^) was in strong linkage disequilibrium with the rs1424597 (r^2^ = 0.85, D′ = 0.99) and also demonstrated strong statistical association with our LIME predictor (p = 2.19 × 10^–6^, Supplementary Table 6) and PE risk (p = 0.007) in MARTHA. Of note, no evidence for association of rs1424597 with gene expression was reported in the GTEx portal.

Accumulating evidence indicates that vascular dysfunction together with a prothrombotic state underlies severe COVID-19 pathophysiology, with respiratory failure linked to microvascular thrombosis in lung^44,45^. With the hypothesis that plasma proteins associated with PE risk would potentially be associated with COVID-19 pulmonary complications, we included an antibody targeting PLXNA4 in a plasma proteomic analysis of 339 samples collected at consecutive time points from 112 hospitalized COVID-19 patients. As indicated in Table 4, plasma PLXNA4 levels tend to slightly decrease with worsened respiratory dysfunction at baseline, patients with RI > 2 having lower PLXNA4 levels (β = −0.107 ± 0.082, p = 0.195) than patients with RI ≤ 2. The longitudinal analysis of all available measurements confirmed this association (β = −0.073 ± 0.033, p = 0.025).

**Table 4:** Association of PLXNA4 plasma levels with Respiratory Index (RI) in COVID-19 patients from the COMMUNITY study.

RI = 0	RI = 1	RI = 2	RI = 3	RI = 4
**Baseline data analysis**				
164.1 [146.7–187.5] N = 42	164.9 [152.4–175.3] N = 45	160.8 [152.8–191.8] N = 17	154.1 [149.8–154.4] N = 3	147.3 [145.5–159.9] N = 3
**Mutiple time point analysis**				
165.4 [147.8–187.7] N = 112	165.3 [151.4–183.3] N = 118	161.6 [151.2–171.6] N = 43	154.4 [151.4–187.5] N = 12	150.4 [140.2–158.4] N = 53

Shown values shown correspond to PLXNA4 median [1st–3rd quartile] of relative MFI levels. Tests for association were performed on log transformed values adjusted for age, sex and body mass index using linear and linear mixed effect models for baseline and multiple time points analyses, respectively. Individuals with RI ≥ 3 tend to exhibit lower log-transformed PLXNA4 levels than individuals with RI ≤ 2, both in the baseline (β = −0.107 ± 0.082, p = 0.195) and multiple time point (β = −0.073 ± 0.033, p = 0.025) analyses .

### Genetics of inconsistent LIME predictions

As shown in Table 2, our ANN/LIME models failed to correctly predict the true VTE outcome in four individuals from the testing set (individuals 10, 11, 12 and 15). First, it is worthy of note that these 4 individuals were all females. Second, the 3 female PE patients wrongly predicted to be DVT (individuals 11, 12 and 15) were all under oral contraceptives (OC) at the time of the PE event (age 45, 35 and 53, respectively), but not individual 10 incorrectly predicted to be PE. While we cannot rule out the possibility that our ANN/LIME models poorly behave in women under OC, we nevertheless sought to investigate whether discordant predictions could be due to genomic outlier individuals harboring very rare disease causing mutations that could make the global ANN/LIME predictions inaccurate, in line with the idea that the discrepancy between (machine learning derived) predicted and observed phenotypes could be a heritable trait^46^. Among these 4 individuals, only two (Individuals 11 and 15) have been sequenced for their whole genome. Sequence data of these two individuals were then scrutinized for candidate rare variants that could explain the VTE phenotype.

Individual 11 is a woman that experienced PE under oral contraceptives (OC) at age 45. Of note, her ten closest neighbors inferred from HPA data were all DVT patients which would likely explain why the derived ANN predicted her a DVT outcome instead of PE. She was not found to harbor any candidate variation in known VTE genes but presented in her genome with 61 very rare coding variants with strong predicted deleteriousness that could be good candidates responsible for the PE event (Supplementary Table 7).

Individual 15 is a woman that had experienced PE at age 53 also under OC. Nine out of 10 of her closest proteomics based neighbors were DVT patients which may also explain why this PE patient was incorrectly predicted to be DVT. This patient was found to carry a very rare nonsynonymous variation (rs121918154; PROC:NM_000312:exon9:c.C814T:p.R272C) in the VTE-associated *PROC* gene. This variation has a minor allele frequency of 0.005% in public database (https://www.ncbi.nlm.nih.gov/snp/rs121918154), is predicted to be deleterious by several bioinformatics tools and have been previously reported in VTE patients with protein C deficiency^47,48^. This variation is located in the last exon of the gene and is predicted to alter splicing regulatory elements^49–51^, which could lead to a deletion of a part of the peptidase S1 domain that is responsible for the cleavage activity of the protein. Of note, this patient exhibited moderately low plasma Protein C levels, 63%, slightly lower than the 65% threshold adopted to declare moderate protein C deficiency^52^.

## Discussion

This work is original in at least three main aspects. First, it is the largest plasma proteomic study with respect to pulmonary embolism in VTE patients. Second, it is to our knowledge the first attempt to deploy ANN methodologies on proteomic data with the aim at identifying new molecular thrombotic players. And finally, the integration of proteomic and genomics data identified *PLXNA4* as a new candidate gene for PE.

This work started with the implementation of an ANN methodology on antibody based affinity proteomics data in relation to PE risk. This ANN was not developed as a tool to be used in clinic for predicting PE risk as 1/one is not 100% certain about the identity of the identified tagged proteins^53^ (further experimental validation would be needed to assess this) and 2/plasma protein levels determined with the antibody suspension bead array are not absolute but relative values depending on the current set of studied antibodies. Rather, we employed this ANN strategy to detect a PE-associated molecular signature that could either reflect nonlinear relationships between investigated proteins or serve as an intermediate surrogate biomarker of an unmeasured variable that could generate new knowledge about the (genetics) mechanisms involved in PE. Our intention is not to claim that the proposed strategy is the panacea but that it can be considered as an appealing strategy compared to others methods. The latter are legion and their exhaustive comparison is out of the scope of the current work. However, preliminary results (see supplementary data) indicate that our ANN strategy performs better on our proteomics data than some popular methodologies such as standard logistic regression and Random Forest.

By conducting a GWAS on the derived PE linear predictor and capitalizing on two case—control samples totaling 467 patients with PE and 1414 patients with DVT, we observed that VTE patients that were homozygotes carriers of the *PLXNA4* rs1424597-A allele were at higher risk of PE.

*PLXNA*4 codes for Plexin A4, which is part of a receptor complex involved in signal transduction of semaphorin 3 signals linked to cytoskeletal rearrangement, inhibiting integrin adhesion^54,55^. It has a role in axon guidance in nervous system development, and genetic variants in PLXN4 have been linked to risk of Alzheimer disease^56,57^. Based on RNA seq data from HPA, FANTOM and GTEx datasets, *PLXNA4* is expressed at medium/high levels in central nervous system, adipose, breast and female reproductive tract tissues, and low levels in a broad range of other tissues (https://www.proteinatlas.org/ENSG00000221866-PLXNA4/tissue), indicating roles outside the nervous system. Despite found in most tissues, based on more recently available data from an integrated analysis of single cell RNA seq data available in public repositories (https://www.proteinatlas.org/humanproteome/celltype), the cell types in which PLXNA4 is expressed in is tissue dependent. For example, in liver it is expressed in endothelial cells together with a low expression in ITO cells but no expression in other cell types found, while in lung, it is found to be expressed in fibroblasts, T cells and granulocytes, but not identified in endothelial cells. In RNA seq data from sorted blood cell populations, PLXNA4 show expression predominantly in plasmacytoid dendritic cells, together with NK cells and low level of expression in some T cell populations. Together, this indicates an organ and cell type dependent regulation of PLXNA4 expression, which could suggest different role in different tissues. Research based on animal studies suggest a role in immunity and immune function, where it has been shown to be a negative regulator of T cell activation^58^. One of the PLXNA4’s ligand, SEMA3, has also been described with a role in endothelial cell function in an autocrine loop, promoting processes involved in vascular remodeling^59^, and also in negatively regulating platelet aggregation^60^. While PLXNA4 thus has been described with a role in processes/pathways of relevance for thrombosis, little is known about *PLXNA4* in pulmonary embolism.

Symptoms of COVID-19, the disease caused by severe acute respiratory syndrome coronavirus 2 (SARS-CoV-2), include fever, fatigue, dry cough and dyspnea. While most individuals experience mild to moderate disease, a proportion progress of infected individuals progress to severe or critical disease with pneumonia, acute respiratory distress syndrome^45^, endotheliopathy leading to microvascular thrombotic complications^61^ contributing to the high incidence of pulmonary embolism observed in COVID-19 patients^62^. By plasma proteomics analysis of 339 samples from 112 hospitalized patients in the COMMUNITY STUDY, we found that plasma levels of PLXNA4 were associated with level of respiratory support needed in critically ill COVID-19 patients.

Nevertheless, we did not identify strong elements supporting a functional role of the intronic rs1424597 polymorphisms or of any other polymorphisms in strong linkage disequilibrium with it. The rs1424597 has recently been observed to associate in trans with whole blood DNA methylation levels at the *ATRIP* locus. However, based on scRNAseq data, the cell type expression profile of ATRIP in different organs and tissues does not match that of PLXNA4 to any notable extent, making difficult any straightforward interpretation of the trans association. Besides, in the FinnGen study (http://r3.finngen.fi/), it has been reported to marginally (p = 4.5 × 10^–3^) associate with pleural conditions that are inflammatory disorders of the lung. Consistent with this observation, we observed a positive correlation between the rs1424597-associated PE predictor and fibrinogen, a well-known inflammatory marker. Additional *PLXNA4* polymorphisms have also been reported to demonstrate strong statistical evidence for association with various lung function markers^63,64^. Altogether, these observations strongly support for a role of *PLXNA4* in lung function and its precise role in the etiology of pulmonary embolism deserve further investigation. Which polymorphisms could be truly responsible for the observed association with PE risk also merits further works as the rs1424597 is likely tagging for functional variant(s)/haplotypes yet to be characterized.

In addition to searching for common polymorphisms that could associate with our ANN based predictor and with PE risk, we also looked for rare variants that could explain the discrepancy between predicted and observed VTE outcome in our testing set. Two out of four patients with discordant predictions in the testing set have been sequenced for their whole genome. Both were female patients that experienced PE under OC. In one of them, we were able to identify a rare VTE causing mutation in *PROC*. It is not our intention to conclude to any general rule about the relevance of searching of rare variants responsible for any discordancy between ANN predictions and observed outcomes. Especially as we observed that the three PE patients wrongly predicted to be DVT were women who developed PE under OC. These observations could suggest that our plasma proteomics ANN derived predictions may not be valid in such subgroups of VTE patients and highlight the challenge to identify general prediction models for complex diseases. Several additional limitations must be addressed.

First, the under sampling strategy we deployed to select patients that will be used in the ANN approach have led to a selection of DVT patients that may no longer be representatives of the whole population of DVT patients as we have discarded DVT patients that are very close, with respect to their biological and proteomic data, to PE patients. As a consequence, the *PLXNA4* locus we identified is likely a susceptibility locus for PE only in a subgroup of VT patients with specific characteristics that need to be identified. Second, no plasma antibody targeting PLXNA4 was available when the screening phase of this work was initiated preventing us from validating further its association with PE. Third, no proteomic data was available in the EOVT study to formally replicate the association of our ANN and LIME predictors with PE risk. Fourth, our GWAS analysis on the ANN derived predictor was performed only in 574 samples which has likely hampered our power to identify genome-wide significant SNPs. We may have then missed additional polymorphisms that could be truly associated with the predictor and could have then helped us to better disentangle its underlying molecular biology. Finally, the moderate sample size of the EOVT study has also likely hampered our power for statistically replicating the association of the lead *PLXNA4* polymorphism with PE. In addition, no information was available in the EOVT study to distinguish isolated PE From DVT + PE which prevented us from further testing whether the association of *PLXNA4* with PE risk was mainly restricted to isolated PE as suggested from the MARTHA results.

In conclusion, by implementing an original artificial neural network methodology integrating plasma proteomics and genetic data, we identified *PLXNA4* as a new candidate susceptibility gene for PE in VTE patients whose precise role in PE etiology deserves further investigations.

## Supplementary Information

Supplementary Information 1.

Supplementary Information 2.

Supplementary Information 3.

Supplementary Information 4.

Supplementary Information 5.

Supplementary Information 6.

Supplementary Information 7.

Supplementary Information 8.

Supplementary Information 9.

Supplementary Information 10.

Supplementary Information 11.

## Data Availability

Proteomics data used in this work are available at https://zenodo.org/record/4280776#.YCEVVeoo-vc.
