# Supplementary figures and images for: An artificial neural network approach integrating plasma proteomics and genetic data identifies PLXNA4 as a new susceptibility locus for pulmonary embolism
Source: Sci Rep. 2021 Jul 7;11:14015. doi: 10.1038/s41598-021-93390-7 (PMC8263618; doi:10.1038/s41598-021-93390-7)

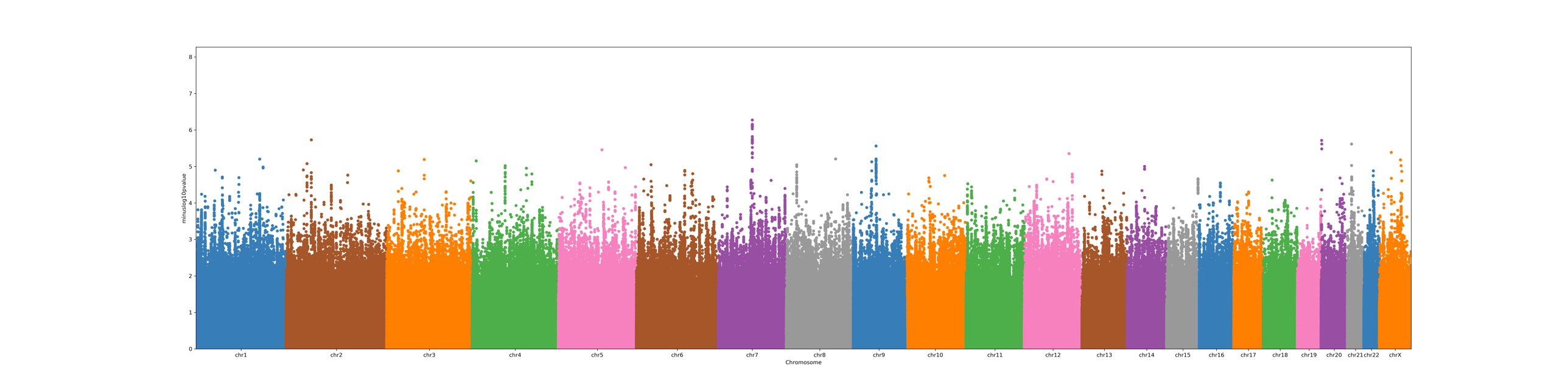

Supplement: Supplementary file 1 — Supplementary Information 1. [file 41598_2021_93390_MOESM1_ESM.png]
