# Supplementary material for: An artificial neural network approach integrating plasma proteomics and genetic data identifies PLXNA4 as a new susceptibility locus for pulmonary embolism
Source: Sci Rep. 2021 Jul 7;11:14015. doi: 10.1038/s41598-021-93390-7 (PMC8263618; doi:10.1038/s41598-021-93390-7)
Supplement: Supplementary file 3 — Supplementary Information 3. [file 41598_2021_93390_MOESM3_ESM.docx]

**Supplementary Table 1 : List of biological phenotypes that have been measured in MARTHA patients**

Factor VIII, Factor XI, von Willebrand Factor, prothrombin time, antithrombin, protein C, protein S, PAI-1, platelets count, red blood cells count, mean platelet volume, mean corpuscular volume, white blood cells count, neutrophils, eosinophil, basophil, lymphocytes, monocytes, fibrinogen,
