# Supplementary material for: An artificial neural network approach integrating plasma proteomics and genetic data identifies PLXNA4 as a new susceptibility locus for pulmonary embolism
Source: Sci Rep. 2021 Jul 7;11:14015. doi: 10.1038/s41598-021-93390-7 (PMC8263618; doi:10.1038/s41598-021-93390-7)
Supplement: Supplementary file 5 — Supplementary Information 5. [file 41598_2021_93390_MOESM5_ESM.docx]

**Supplementary Table 3 Characteristics of the EOVT study**

|  | DVT | PE |
| --- | --- | --- |
| N | 196 | 143 |
| Age at first VTE | 35.5 (9.46) | 36.6 (9.95) |
| Female sex | 109 (56%) | 58 (40%) |
| FV Leiden (rs6025) heterozygotes | 34 (17%) | 25 (17%) |

DVT : Deep Vein Thrombosis ; PE : Pulmonary Embolism ;

Data shown correspond to mean (standard deviation) and count (percentage) for continuous categorical variables, respectively
