# Supplementary material for: An artificial neural network approach integrating plasma proteomics and genetic data identifies PLXNA4 as a new susceptibility locus for pulmonary embolism
Source: Sci Rep. 2021 Jul 7;11:14015. doi: 10.1038/s41598-021-93390-7 (PMC8263618; doi:10.1038/s41598-021-93390-7)
Supplement: Supplementary file 6 — Supplementary Information 6. [file 41598_2021_93390_MOESM6_ESM.docx]

**Supplemental Table 4 Characteristics of the COMMUNITY study**

|  | All | Female | Male |
| --- | --- | --- | --- |
| N | 112 | 41 | 71 |
| Age (years) ; mean (SD) | 59.32 (14.98) | 52.92 (17.72) | 63.01 (11.77) |
| BMI (Kg/m2) ; mean (SD) | 28.73 (6.16) | 29.53 (7.96) | 28.18 (4.86) |
|  |  |  |  |
| Lenth of stay (days) ; mean (SD) | 9.04 (8.36) | 8.58 (9.38) | 9.31 (7.82) |
|  |  |  |  |
| **Max Respiratory support** |  |  |  |
| no respiratory support (RI0) | 38 | 17 | 21 |
| ≤5 L of oxygen on nasal cannula or mask (RI1) | 42 | 17 | 25 |
| > 5 L of oxygen on nasal cannula or mask (RI 2) | 15 | 5 | 10 |
| Non invasive ventilation (NIV)/ High-flow nasal cannula (HFNC) (RI 3) | 5 | 0 | 5 |
| intubation (RI4)) | 12 | 2 | 10 |
|  |  |  |  |
| mortality < 1 month (%) | 13 | 1 | 12 |
|  | 11,60% | 2,40% | 16,90% |
|  |  |  |  |
| LMWH prophylactic administration | 86 (76.8%) | 33 (80.50%) | 53 (74.65%) |
|  |  |  |  |
| Acute PE | 5 | 1 | 4 |
| Acute DVT | 1 | 0 | 1 |
|  |  |  |  |
| **Smokers** |  |  |  |
| No | 52 | 24 | 28 |
| Yes | 3 | 0 | 3 |
| Previous | 28 | 6 | 22 |
| Unknown | 29 | 11 | 8 |
|  |  |  |  |
| **Clinical lab values** |  |  |  |
| D-Dimer [mg/L FEU](n=76)* | 2.61 (4.48) | 2.44 (3.72) | 2.72 (4.91) |
| Q1-Q3 | (0.795-2.325) | (0.83-1.96) | (0.675-3.05) |
|  |  |  |  |
| CRP [ mg/L] (n=108)** | 122.25 (88.66) | 101.4 (81.31) | 134.51 (91.07) |
| Q1-Q3 | (61.75-170) | (35-147.25) | (75-179.5) |

* D-dimer measurements were available in 29 females and 47 males.

** CRP measurements were available in 40 females and 68 males
