# Supplementary material for: An artificial neural network approach integrating plasma proteomics and genetic data identifies PLXNA4 as a new susceptibility locus for pulmonary embolism
Source: Sci Rep. 2021 Jul 7;11:14015. doi: 10.1038/s41598-021-93390-7 (PMC8263618; doi:10.1038/s41598-021-93390-7)
Supplement: Supplementary file 10 — Supplementary Information 10. [file 41598_2021_93390_MOESM10_ESM.docx]

**Structure of the implemented artificial neural network.**

The neural network was trained using keras open source library in Python using the following parameters:

| Number of hidden layers | 2 |
| --- | --- |
| Number of nodes in first hidden layer | 395 |
| Number of nodes in second hidden layer | 128 |
| Activation function at hidden layers | ReLU |
| Activation function at output layer | Softmax |
| Learning rate | 0.01 |

**Logistic regression analysis of the 376 HPAs in relation to PE risk in the 1388 MARTHA participants**

After adjusting for age and sex, the strongest association was observed with the HPA042431 (p = 0.001). This compares to the association p-value of 1.3 10^-14^ observed with the LIME predictor.

The top 10 most significant HPAs identified by the logistic regression as shown below : Note that , among the top

| HPA | Association P-value with respect to PE risk |
| --- | --- |
| HPA042431 | 0.0010440706 |
| HPA063446 | 0.0011725939 |
| HPA040815 | 0.0018919429 |
| HPA052272 | 0.0018985986 |
| HPA044659 | 0.004465882 |
| HPA011143 | 0.0061332159 |
| HPA020741 | 0.0073193354 |
| Bsi0270 | 0.0080629771 |
| Bsi0731 | 0.0086508004 |
| HPA003891 | 0.010570137 |

Note that among these top 10 HPAs identified by logistic regression analysis, only HPA040815 was selected (rank 15th) among the 20 most important HPAs identified by the ANN strategy (Figure 3).

**Preliminary comparison with a classical Random Forest methodology**

We here provide a preliminary study comparing the performances of our ANN strategy and of a standard random forest algorithm on MARTHA data. For the latter method, we used scikit learn API to build a random forest with 100 trees (Number of trees as suggested in https://link.springer.com/chapter/10.1007/978-3-642-31537-4_13).We applied the same data processing steps as the one used for our ANN based strategy.

The application of Random Forest methods led to an AUC of 0.62 that compares to 0.79 for our ANN model


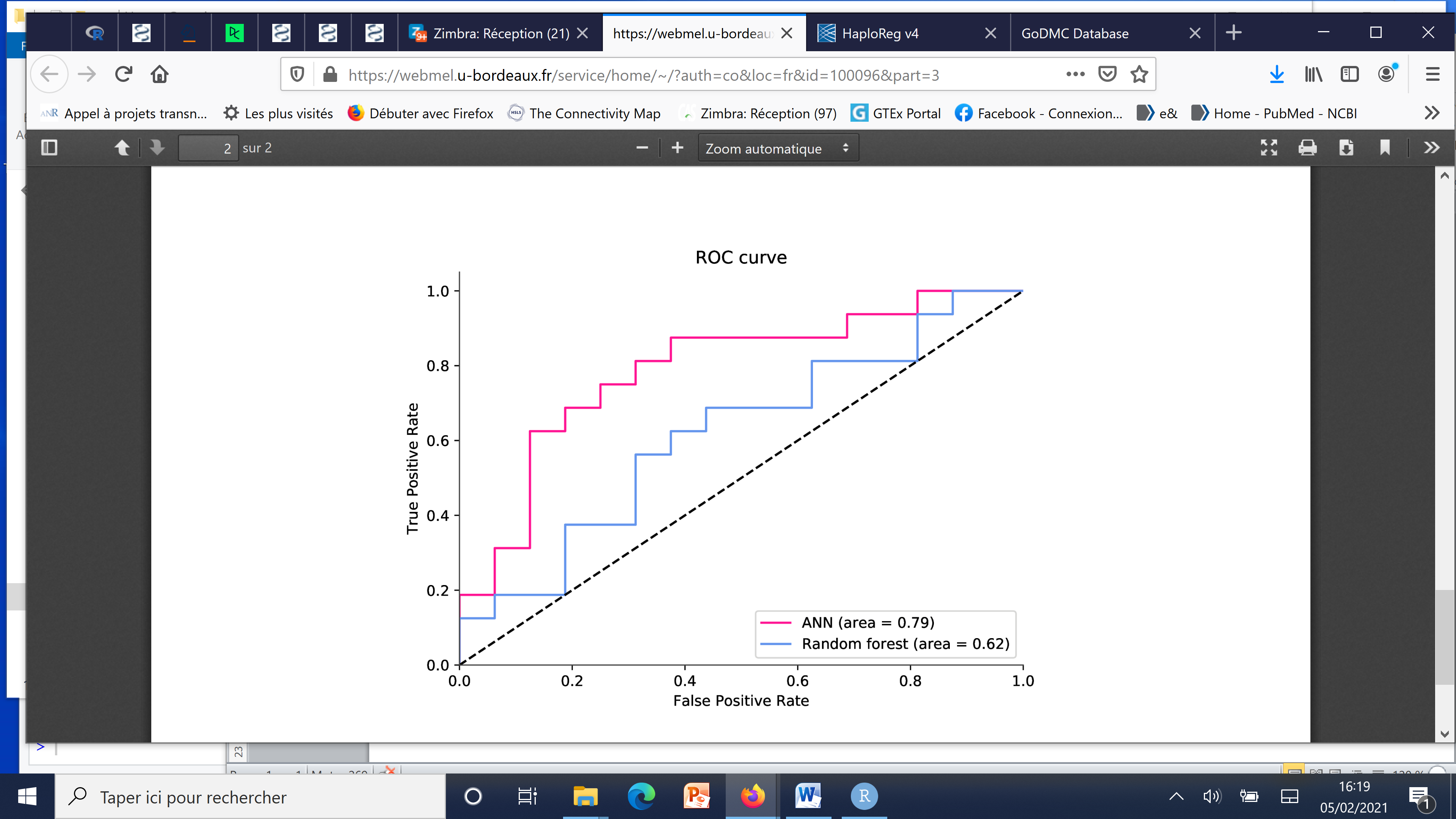


. Other performance metrics are shown in the table below

|  | ACC | F1 score | | Recall | | Precision | |
| --- | --- | --- | --- | --- | --- | --- | --- |
|  |  | DVT | PE | DVT | PE | DVT | PE |
| Random Forest | 0.62 | 0.75 | 0.25 | 0.90 | 0.17 | 0.64 | 0.50 |
| ANN | 0.75 | 0.82 | 0.60 | 0.90 | 0.50 | 0.75 | 0.75 |
