# Supplementary material for: An artificial neural network approach integrating plasma proteomics and genetic data identifies PLXNA4 as a new susceptibility locus for pulmonary embolism
Source: Sci Rep. 2021 Jul 7;11:14015. doi: 10.1038/s41598-021-93390-7 (PMC8263618; doi:10.1038/s41598-021-93390-7)
Supplement: Supplementary file 11 — Supplementary Information 11. [file 41598_2021_93390_MOESM11_ESM.docx]

**Supporting information captions**

**Supplementary Figure 1** Manhattan plot describing the results of the genome wide association study on the LIME predictor

**Supplementary Figure 2** Graphical representation of the ANN dataset projected on the first two principal components derived from standard principal components analysis , t-SNE and UMAP techniques

**Supplementary Table** 1 List of biological phenotypes that have been measured in MARTHA patients

**Supplementary Table 2** List of Human Protein Atlas antibodies measured in MARTHA patients

**Supplementary Table 3** Brief characteristics of the EOVT participants

**Supplementary Table 4** Characteristics of the COMMUNITY study

**Supplementary Table 5** Correlation between LIME PE predictor and biological traits available in MARTHA participants used for building the ANN

**Supplementary Table 6** Main statistical associations (p< 1 x 10^-5^) observed in the GWAS on LIME predictor

**Supplementary Table 7** Rare coding variants identified by whole genome sequencing in individual 11.
